# Aridity and drought: distinct concepts often conflated in ecological research

**DOI:** 10.1111/nph.71082

**Published:** 2026-03-13

**Authors:** Alan K. Knapp, Abbey Chatwin, Jenna E. Gardner, Anna Hall, Adriana Jacobi, Scott Otto, J. Alexander Siggers, Joseph G. Toman, E. Greg Tooley, Robert J. Griffin‐Nolan

**Affiliations:** ^1^ Department of Biology and Graduate Degree Program in Ecology Colorado State University Fort Collins CO 80523 USA; ^2^ Department of Forest and Rangeland Stewardship Colorado State University Fort Collins CO 80523 USA; ^3^ Department of Soil and Crop Sciences and Graduate Degree Program in Ecology Colorado State University Fort Collins CO 80523 USA; ^4^ Department of Biological Sciences California State University Chico CA 95929 USA

**Keywords:** aridity, conservative vs acquisitive, drought, drought strategies, functional traits, leaf economic spectrum, water limitation

## Abstract

As well‐established drivers of ecological pattern and process, aridity and drought are linked by a common focus on water limitation – with both aridity and especially drought projected to increase in extent and severity with climate change. There is a long history of assessing plant adaptations to drought through the lens of aridity. Consistent with this history, trait‐based studies of drought typically frame their hypotheses around how plants, communities, and ecosystems vary along spatial aridity gradients. However, aridity, as a long‐term, water‐limited climatic state, differs fundamentally from drought, which is an anomalous but discrete period of severe water limitation. Here, we ask whether patterns observed along aridity gradients should conceptually underpin how plants are hypothesized to respond to drought. We briefly review literature that either supports or challenges the use of traits that vary along aridity gradients to predict responses to drought. We then highlight reasons why plants and ecosystems might be expected to respond differently to aridity and drought. We conclude that the conflation of aridity with drought may hamper progress in understanding the ecological consequences of globally increasing water limitation. Advancing the field of drought ecology would be better served by testing hypotheses arising from alternative frameworks.

## Aridity, drought, and climate change

For decades, increasing greenhouse gases and a warming atmosphere have been predicted to alter the hydrological dynamics of our planet (e.g. Rind *et al*., 1990; Loaiciga *et al*., 1996; Trenberth, 1998, 2011; Easterling *et al*., 2000; Trenberth *et al*., 2003). Today, the scientific consensus is that such alterations are well underway (Chiang *et al*., 2021; IPCC, 2021; Yang *et al*., 2021; Feldman *et al*., 2024) with substantial evidence pointing to a future of increasing aridity (Huang *et al*., 2016; Lian *et al*., 2021; D. Luo *et al*., 2023; but see Zhou & Yu, 2025), and more frequent and severe droughts in many parts of the world (Dai, 2011; Trenberth *et al*., 2014; Ault, 2020; Vicente‐Serrano *et al*., 2022; Gebrechorkos *et al*., 2025; L. Chen *et al*., 2025). These hydrological manifestations of climate change are especially alarming given that a large proportion of the global land surface is already water‐limited for key carbon cycle processes (e.g. primary productivity; Churkina & Running, 1998; Anav *et al*., 2015). Unsurprisingly, reports of increasing aridity and drought have motivated extensive research aimed at assessing, quantifying, and better understanding their ecological consequences (e.g. Slette *et al*., 2019; Berdugo *et al*., 2020; Knapp *et al*., 2024; Smith *et al*., 2024; Tariq *et al*., 2024; Wilder *et al*., 2025).

**Fig. 1:**
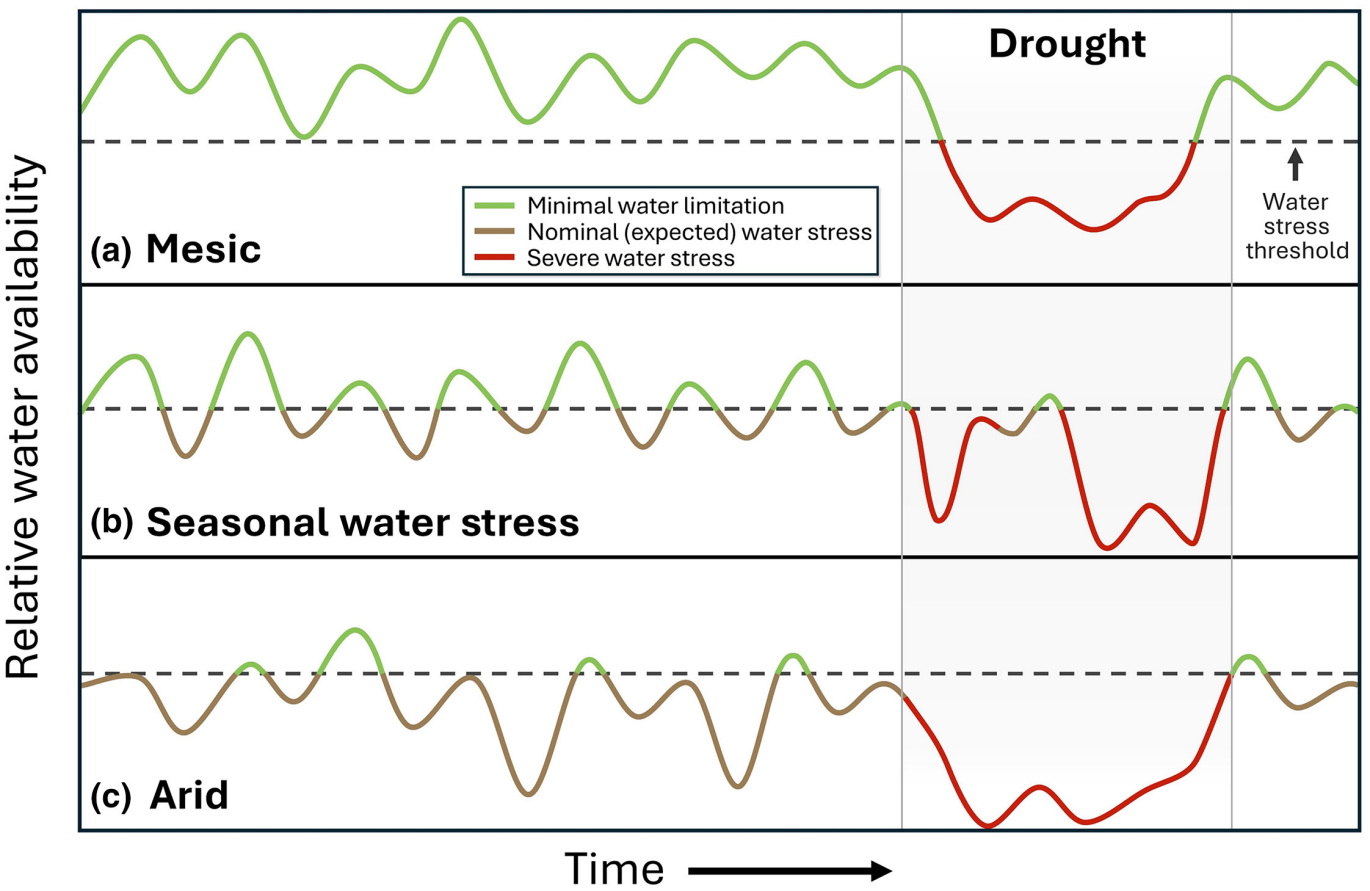
Conceptual representation of how different ecosystems typically experience water limitation (ordered top to bottom from (a) mesic to (b) seasonally dry to (c) arid ecosystems) in contrast with drought periods (right), which are anomalous and distinct. Note that the time scales (*x*‐axis) are relative and differ for each panel. Minimal water stress periods (above the dashed lines) are when water stress is absent (or relatively low) and most plant growth occurs, whereas nominal water stress periods (below the dashed line) are when growth is water‐limited to some degree. Drought (lines within the shaded area) is defined as an anomalous period of reduced water availability that usually leads to more severe water limitation than normal in plants/ecosystems. Note that water stress and growth limitation may occur during nondrought periods (in all but the most mesic ecosystems, (a)), but relative to an ecosystem's typical state, drought is a discrete and relatively rare period of unusually severe water shortage. In many ecosystems such as those with Mediterranean climates (b), there are predictable and recurring periods of water limitation every year (e.g. the ‘summer dry season’). Even though these periods of water limitation can be severe and are discrete, they are also regular features of the climate and thus differ fundamentally from drought. In arid ecosystems (c), water stress is the most common state. But note that drought can also occur in arid ecosystems, based on the definition described previously, often due to an anomalous duration of low water availability. We argue that even if the severity of water stress is similar during periods or normal or expected water stress vs periods of drought, how plants and ecosystems respond to seasonal or chronic periods of water stress may not be informative for understanding responses to drought.

Both aridity and drought are well‐established drivers of ecological patterns and processes (Noy‐Meir, 1973; Maestre *et al*., 2016; Slette *et al*., 2019; Berdugo *et al*., 2020; Hoover & Smith, 2025) and while they share a focus on water limitation, aridity and drought differ in other key dimensions. Briefly, aridity is a long‐term climatic state of water scarcity that is typically caused by low precipitation and high atmospheric evaporative demand (Vicente‐Serrano *et al*., 2024). Although drought can be defined from many perspectives (Crausbay *et al*., 2017; Slette *et al*., 2019), ecological drought is generally considered to be an anomalous period of low water availability of sufficient duration/severity (Felton *et al*., 2021; Lisonbee *et al*., 2021) to potentially alter ecosystem structure and function (Knapp *et al*., 2024). An essential distinction between these two concepts is that aridity is a normal or expected climatic state, whereas drought is an anomalous period within a given climate (Fig. 1). Critically, drought can occur in any climate, affecting both arid and humid ecosystems (Spinoni *et al*., 2014). However, there is typically a recovery period after drought (Gutschick & BassiriRad, 2003; Vilonen *et al*., 2022) with unique plant mechanisms enabling recovery (e.g. Illouz‐Eliaz *et al*., 2025). But plants do not recover from aridity. In other words, aridity is not a continuous drought.

Below, we argue that ecological studies of drought frequently, albeit unintentionally, blur the distinction between aridity and drought. This conflation of drought and aridity can be attributed, in part, to drought seldom being explicitly defined in ecological studies. ‘Drought’ is a term often used colloquially to describe dry conditions or periods of water stress (Slette *et al*., 2019). Arid and semiarid ecosystems experience such dry conditions much of the time. Thus, it is not surprising that our understanding of how plants and ecosystems cope with aridity (or annually recurrent periods of water stress) often conceptually underpins the hypotheses we test when drought is studied. This is particularly common in trait‐based approaches to the study of drought impacts on plants and ecosystems. Our concern is that progress toward understanding the ecological consequences of globally increasing aridity and drought may be hampered by the conflation of these two concepts.

## A brief review of research linking aridity, drought, and plant traits

Ecologists have historically looked to arid ecosystems, or those with seasonally predictable periods of severe water stress (e.g. the summer dry season in Mediterranean systems; Fig. 1), to study drought acclimation and adaptation in plants (e.g. Laude, 1953; Oppenheimer, 1960; Noy‐Meir, 1973; Fischer & Turner, 1978; Ehleringer *et al*., 1999; Reynolds *et al*., 1999; Chaves *et al*., 2003), a practice that continues today (e.g. Chomentowska *et al*., 2025; Rimer & McAdam, 2025). Indeed, aridity has been explicitly argued to be ‘an environmental filter selecting for traits related to key plant strategies to cope with severe and often unpredictable drought’ (Nunes *et al*., 2017; see also Moreira *et al*., 2025). Such an approach seems reasonable as morphological, anatomical, and physiological traits for coping with chronic low water availability might be expected to provide similar benefits to plants under more extreme drought conditions (Sandquist & Ehleringer, 1997; Kimball *et al*., 2017; Welles & Funk, 2021; Kaproth *et al*., 2023; Metz & Tielbörger, 2023; Knapp *et al*., 2024). It is also well‐established that plant traits vary predictably, and often in a coordinated manner, across environmental gradients, habitats, and disturbance regimes – including gradients of water availability (Reich *et al*., 1999; Niinemets, 2001; Wright *et al*., 2001, 2004; Ackerly *et al*., 2002; Wright & Westoby, 2002; K. Sun *et al*., 2025). Accordingly, although dryland plant communities exhibit high trait diversity, what are considered ‘drought‐adapted traits’ such as small, sclerophyllous leaves with low specific leaf area (SLA, leaf area per unit mass), high root : shoot ratios, and succulence tend to be over‐represented in arid environments (e.g. Schenk & Jackson, 2002; Wright *et al*., 2004, 2017; Maestre *et al*., 2021).

Traits, historically referred to as ‘attributes’ or ‘characteristics’ of plants or vegetation types (Lerdau *et al*., 2023), have been defined in many ways (Dawson *et al*., 2021; de Bello *et al*., 2025). Terminology aside, traits have long been used by ecologists to better understand plant adaptations, responses to disturbance, and the spatial and temporal dynamics of species and populations (Harper, 1967; Bazzaz, 1979; Noble & Slatyer, 1980; Weiher *et al*., 1999; Lerdau *et al*., 2023). Trait‐based research is successful because traits not only influence individual plant responses, fitness, and demography (Violle *et al*., 2007; Adler *et al*., 2014) but also be directly related to ecosystem functions and/or mediate environmental impacts on ecosystems (Lavorel & Garnier, 2002; Asner *et al*., 2016; Funk *et al*., 2017; Blouin *et al*., 2025; de Bello *et al*., 2025).

More than 40 years ago, Grime (1974, 1979) proposed one of the more influential and synthetic trait frameworks known as the competitor‐stress‐ruderal theory (Grace, 1991; Fridley *et al*., 2023). This framework sought to explain the range of plant ecological strategies across community types emphasizing trade‐offs between stress tolerance (including water limitation) and resource capture. Later, the Leaf Economic Spectrum (LES) was proposed, which identified a trade‐off between traits related to rapid growth rates (e.g. high leaf nitrogen content, SLA, and photosynthetic rates) and leaf lifespan (Reich *et al*., 1999, 2003; Wright *et al*., 2004). The LES focused on understanding how sets of leaf traits related to resource investment were coordinated as plant strategies, and how these might be shaped by selective forces, physical constraints, and the environment, including resource availability and climate (nutrients, temperature, and water, e.g. Westoby & Wright, 2006; Shipley *et al*., 2006; Donovan *et al*., 2011). Although originally focused on leaf traits, the framework was later expanded (Reich, 2014) to encompass an acquisitive‐conservative (fast‐slow) spectrum of traits, with evidence of convergence across leaf, root, and stem traits that define ecological strategies regardless of the limiting resource (light, N, or water). This implies that conservative growth traits found in low nutrient environments are expected to be advantageous for coping with low water availability as well. Thus, drought tolerance traits are expected to be associated with a resource‐conservative strategy, whereas ‘acquisitive traits’, associated with high plant growth rates and increased productivity, should lead to increased drought vulnerability (Reich, 2014; Ramírez‐Valiente & Cavender‐Bares, 2017) unless drought can be escaped or avoided (Volaire & Norton, 2006; Kooyers, 2015). This concept is now firmly entrenched in the literature, exemplified by the assertion that ‘it has been repeatedly proved that “fast species”… are more vulnerable to drought compared with “slow” species’ (Wang *et al*., 2025) and thus is the conceptual basis for much drought research (e.g. Harrison *et al*., 2015; Bär Lamas *et al*., 2016; Costa‐Saura *et al*., 2016; Greenwood *et al*., 2017; Nunes *et al*., 2017; Carvajal *et al*., 2019; Welles & Funk, 2021; Xu *et al*., 2022; Wang *et al*., 2023; Benson *et al*., 2025; Künzi *et al*., 2025; Y. Chen *et al*., 2025).

Although ecologists have readily adopted the fast‐slow framework to underpin hypotheses about trait responses to drought, both within and among species (Jung *et al*., 2014; Lopez‐Iglesias *et al*., 2014; Kimball *et al*., 2016; Balachowski & Volaire, 2018; Griffin‐Nolan *et al*., 2019; Welles & Funk, 2021; Kramp *et al*., 2022; Iozia *et al*., 2023; Jardim *et al*., 2025), it is important to emphasize that the conservative‐acquisitive spectrum of traits originated from trait variation observed across resource gradients (including moisture availability, Reich, 2014), but not in direct response to drought. Thus, it is worth asking whether trait responses to aridity gradients can provide mechanistic insights relevant to understanding how plants, and the traits that drive ecosystem processes, respond to drought (de Bello *et al*., 2025) – given that aridity and drought are related but fundamentally different concepts. This question goes beyond the original leaf trait focus of the LES (Reich *et al*., 1999; Wright *et al*., 2004). For example, plant hydraulic traits have been the focus of much drought research because they are expected to be directly linked to drought tolerance and survival, especially for trees (Choat *et al*., 2007, 2012; Anderegg *et al*., 2016; Hammond *et al*., 2019; Brodribb *et al*., 2020; Ocheltree *et al*., 2020; Martínez‐Vilalta *et al*., 2023; Rimer & McAdam, 2025; Tijerín‐Triviño *et al*., 2025). But even here, variation in hydraulic traits observed along dry to wet gradients is often the conceptual basis for predictions of drought tolerance (Kursar *et al*., 2009; Májeková *et al*., 2021; Kerr *et al*., 2022; Martínez‐Vilalta *et al*., 2023; Nosenko *et al*., 2025) – with the assumption that hydraulic traits characteristic of more arid ecosystems will have value for understanding and predicting tolerance to drought.

## What is the evidence that aridity gradients inform drought responses?

Drought studies that explicitly test hypotheses based on how plant traits respond to aridity gradients, based on the conservative‐acquisitive trait framework, are quite numerous (as discussed earlier). Many of these studies show the expected response – that plant traits respond similarly to both increasing aridity along spatial gradients and to drought (e.g. Hallik *et al*., 2009; Kursar *et al*., 2009; Bartlett *et al*., 2014; Blumenthal *et al*., 2020; Xu *et al*., 2022; Aspinwall *et al*., 2023; Nosenko *et al*., 2025; Pan *et al*., 2025; Perez‐Martinez *et al*., 2025). Of the wide range of traits assessed, those that vary over short time frames and reflect carbon and water fluxes (e.g. transpiration, stomatal conductance, and photosynthetic potential) tend to vary most consistently along aridity gradients and also help explain variation in plant growth responses to drought (Aspinwall *et al*., 2023). For example, across a diversity of species with distinct LES traits (e.g. leaf N and SLA), it was water conservation traits (e.g. low transpiration) that best explained variation in drought survival (Funk *et al*., 2024). But there are many studies that also support consistent responses of LES traits (leaf N and SLA) to both aridity and drought (e.g. Hallik *et al*., 2009; Blumenthal *et al*., 2020), as well as other traits related to plant resource investment (e.g. wood density). Eucalyptus trees, for example, from more arid climates tended to have higher wood density and this trait best explained sensitivity of individuals to drought treatments (Perez‐Martinez *et al*., 2025).

By contrast, many studies suggest that drought (natural or experimental) can elicit trait responses that differ strongly from patterns observed along aridity gradients (e.g. Wellstein *et al*., 2017; Griffin‐Nolan *et al*., 2019, 2023; Carvalho *et al*., 2022; Bushey *et al*., 2023; Funk *et al*., 2024; Künzi *et al*., 2025; Ramos‐Muñoz *et al*., 2025; Yan *et al*., 2025). For example, species with conservative LES traits (low SLA and leaf N) may be expected to be more tolerant of drought given the general association of these traits with increasing aridity, yet several studies report that the most drought resistant species or populations are likely to have acquisitive traits (high SLA and turgor loss points; Ramírez‐Valiente & Cavender‐Bares, 2017) and fast growth rates (Ocheltree *et al*., 2016) contrary to expectations (Funk *et al*., 2024; Huang *et al*., 2025). One potential explanation for this is that species characterized by fast‐growth traits can more effectively compete for available water, and in some cases complete their life cycle, before drought progresses in severity.

Beyond individual studies, when large numbers of species responses have been reported, or numerous studies have been compiled and synthesized, trait variation described by the conservative‐acquisitive continuum predicts drought responses inconsistently at best (e.g. Craine *et al*., 2013; Maréchaux *et al*., 2015, 2020; Griffin‐Nolan *et al*., 2018; Martínez‐Vilalta *et al*., 2023; Funk *et al*., 2024; Pan *et al*., 2024). For example, across more than 70 species of tropical trees, leaf‐level drought tolerance (quantified as leaf turgor loss point) was not correlated with traits commonly associated with the conservative‐acquisitive trait spectrum (e.g. SLA, leaf nitrogen, and wood density; Maréchaux *et al*., 2015). Similarly, in a review of > 500 water manipulation studies, there was no consistent pattern (positive or negative) between traits (SLA and leaf N, as well as maximum plant height, seed mass, and wood density) and water availability as would be expected based on aridity gradients (Griffin‐Nolan *et al*., 2018). Liu *et al*. (2021) assessed the response of SLA in > 1000 grassland species and found that this commonly measured trait varied inconsistently along aridity gradients yet decreased in response to simulated drought. They concluded that responses of SLA along broad environmental gradients, representing long‐term adaptation, were divergent from short‐term responses to drought. Finally, Choat *et al*. (2012) reported that despite hydraulic traits being strongly related to mean annual precipitation, hydraulic safety margins for coping with drought were largely independent of mean annual precipitation. It has been argued that while hydraulic traits may vary with aridity, it is their capacity for plastic adjustment that may determine how plants respond to increasing drought frequency and intensity, with recent evidence suggesting this capacity is limited (Ramírez‐Valiente *et al*., 2025).

Finally, at the ecosystem level, a global meta‐analysis (>300 experiments; Hu *et al*., 2025) tested the hypothesis that biomass production in systems dominated by species with conservative traits would be more drought resistant (i.e. their responses to drought would be reduced) than ecosystems with more acquisitive traits. Instead, they found that ecosystems dominated by slow‐growing species were more sensitive to drought and that mycorrhizal type and the presence of N‐fixers were stronger predictors of biomass sensitivity to drought (Liu *et al*., 2024). These results are consistent with the preponderance of evidence that arid ecosystems, presumed to be dominated by species with conservative traits, are usually more sensitive to drought (natural or experimentally imposed) than mesic systems (Vicente‐Serrano *et al*., 2013; Du *et al*., 2018; Knapp *et al*., 2024). If the plant traits that consistently increase with aridity do not reduce drought sensitivity, it calls into question the assumption that variation in these traits across resource gradients should be used to underpin hypothesized drought responses.

## Why traits that vary across aridity gradients might not be predictive of drought responses?

The lack of alignment between predictions from the conservative‐acquisitive trait framework and drought responses, as well as the generally weak trait–environment relationships observed overall, have been attributed to a wide array of ecological, evolutionary, and methodological factors (Cavender‐Bares & Bazzaz, 2000; Reich *et al*., 2003; Wright *et al*., 2004; He *et al*., 2010; Lintunen *et al*., 2013; Ramírez‐Valiente & Cavender‐Bares, 2017; Griffin‐Nolan *et al*., 2018; Maréchaux *et al*., 2020; Anderegg, 2023; Martínez‐Vilalta *et al*., 2023; Funk *et al*., 2024; Sandel & Griffin‐Nolan, 2024; Benson *et al*., 2025; de Bello *et al*., 2025; Pan *et al*., 2025; Ramos‐Muñoz *et al*., 2025). Here, rather than focus on why responses to aridity and drought diverge from expectations, we pivot and highlight three reasons (nonexclusive hypotheses) why we should not *a priori* expect drought‐driven responses in plant traits, individuals, communities, or ecosystems to be related to trait variation observed along water availability gradients.

**Fig. 2:**
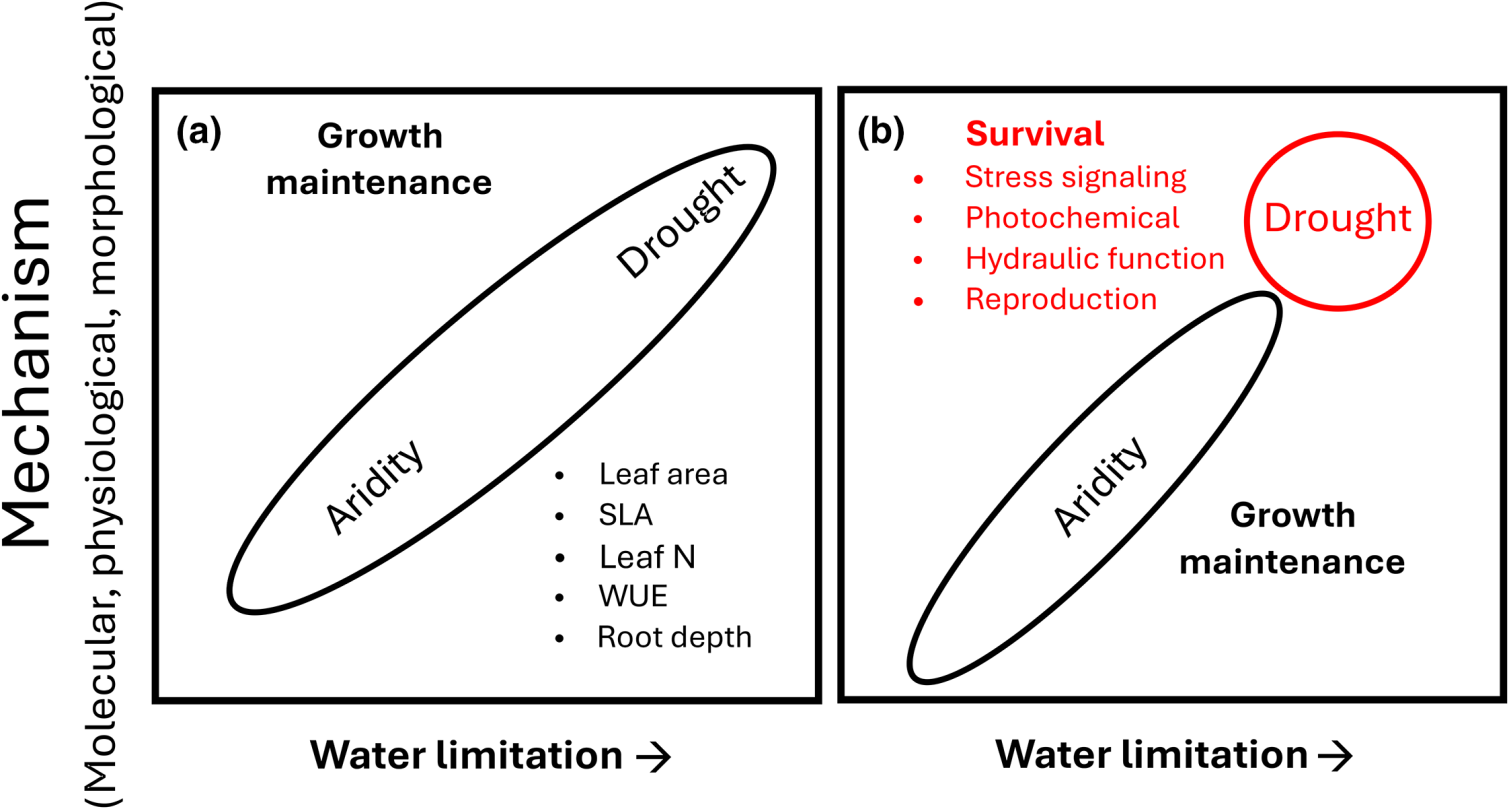
Conceptual representation of how plant strategies (suites of plant traits and mechanisms depicted in multidimensional space) can vary as water limitation increases – from a normal state of aridity to an extreme period of drought. (a) Viewed through the lens of growth maintenance, plants and trait values may respond progressively and consistently to increasing water limitation. Note however that there is evidence that a nonlinear (hump‐shaped) relationship exists between increasing water limitation and some traits (Sandel & Griffin‐Nolan, 2024). Alternatively, (b) there are distinct sets of mechanisms and traits related primarily to survival (Volaire, 2018; Vadez *et al*., 2024; Volaire *et al*., 2026) that only (or uniquely) respond to drought. We recognize that, for example, some stress response pathways and photochemical protective mechanisms that increase drought survival (Chaves *et al*., 2003) may also impact growth, but these are often only elicited under extreme water stress (drought) after growth has ceased. This perspective is adapted from how plants respond to warm temperatures vs heat stress (Kan *et al*., 2023).

First, expecting traits that shift directionally with aridity to be predictive of drought responses implies that the trade‐offs and mechanisms plants invoke in response to increasing water limitations are the same for both aridity and drought (Fig. 2a). From the perspective of growth maintenance, this may be true. Certainly, species from arid ecosystems must, by definition, be adapted to persist under various degrees of water limitation. But as noted previously, aridity, as a climatic state of water limitation, differs fundamentally from drought, as an anomalous period of more severe water limitation. Thus, it is likely that many of the mechanisms and traits correlated with increasing aridity differ from those conferring drought tolerance and/or survival. Indeed, it is well‐established that different intensities of the same plant stress often act through very different pathways (Verslues *et al*., 2023). Furthermore, when drought is viewed as an extreme climatic event (Smith, 2011), species/populations can be pushed beyond critical thresholds and into environmental conditions that differ from their ecological requirements. This can trigger unique evolutionary processes and render traits related to aridity less informative about drought responses (Heinen *et al*., 2025). For example, survivors of a mortality‐inducing extreme drought were reported to have higher not lower SLA and more exploitive root traits (Lloret *et al*., 2016), contrary to expectations. Overall, this may explain why the expected trade‐offs between growth rate and aridity tolerance that underpin the fast‐slow trait spectrum are not always evident when hydraulic and carbon traits are assessed under drought conditions (Huang *et al*., 2025).

An alternative hypothesis is that plants utilize a set of distinct mechanisms to cope with and survive drought (Volaire *et al*., 2026). Agricultural plant breeders (Vadez *et al*., 2024) have recognized this distinction by parsing traits important for growth maintenance under dry conditions vs survival under drought conditions (Fig. 2b). Indeed, distinctions between long‐term responses to water deficits vs short‐term stress responses to drought are well‐known from molecular and physiological studies (Chaves *et al*., 2003). For example, flowering time is a phenological trait that can shift in response to various plant stresses, including drought, with clear ecological and evolutionary consequences (Jordan *et al*., 2015). Stress‐regulated flowering typically occurs only under extreme conditions and is triggered by distinct signaling mechanisms that alter floral pathways (Kazan & Lyons, 2016); thus, flowering as a drought response may differ markedly from how flowering times shift along a spatial aridity gradient (e.g. Kigel *et al*., 2011). It is perhaps most likely that there are shared (constitutive) and distinct (stress induced) mechanisms and traits for coping with aridity and drought, respectively, with many traits that conceptually bridge growth maintenance and survival (Fig. 2b; anisohydry vs isohydry; McDowell *et al*., 2008). This may explain, in part, why it is not uncommon for drought to elicit trait responses associated with both acquisitive and conservative strategies simultaneously (Berger & Ludwig, 2014; Welles & Funk, 2021; Blanco‐Sánchez *et al*., 2023; Asadyar *et al*., 2024; Ramos‐Muñoz *et al*., 2025).

By analogy, mechanisms of plant responses to chronically cool or warm growth temperatures are often distinct from those that confer freezing tolerance (Fontes *et al*., 2025) or reduce the impacts of heat waves (e.g. production of heat shock proteins; Kan *et al*., 2023). In other words, there are fundamentally different selective pressures and traits associated with plant responses to normal vs extreme temperatures. Heatwaves can be viewed as conceptually similar to drought in that they are short‐term, anomalous deviations in temperature that can have dramatic effects on plants, with postheatwave legacies that propagate to the ecosystem level (e.g. Breshears *et al.*, 2021; Kwon *et al*., 2025). However, in crop species, the mechanisms (and traits) that enable survival during heat waves (Sinha *et al*., 2025) differ from those that maintain yield (growth) under warm temperatures (e.g. Bernacchi *et al*., 2025; Cavanagh & Matthews, 2025). There are clear temperature thresholds in both native and crop species that invoke very different physiological mechanisms and responses (e.g. Scafaro *et al*., 2021). For example, in many species, stomatal conductance may decrease as temperatures warm (and vapor pressure deficits increase) to conserve water, but stomata may remain open during heat waves to cool leaves via transpiration (Teskey *et al*., 2015; López *et al*., 2022; Guo *et al*., 2025). Ecological mechanisms that determine plant community and trait responses to increasing temperatures may also show nonlinear behaviors as temperatures become extreme (e.g. Shen *et al*., 2025). Such thresholds and nonlinear responses to increasing water stress are less appreciated (Sandel & Griffin‐Nolan, 2024), but likely as important as water limitation becomes more extreme (Vadez *et al*., 2024).

Finally, as with aridity and drought, there is inconsistent evidence that long‐term climate (mean annual temperature) is a good predictor of tolerance to heat waves or warming in general (O'Sullivan *et al*., 2017; Zhu *et al*., 2018; Lancaster & Humphreys, 2020; Perez & Feeley, 2021; Slot *et al*., 2021; Andrew *et al*., 2022; Didion‐Gency *et al*., 2025; Dobson & Zarnetske, 2025; Garen & Michaletz, 2025). Indeed, similar to evidence that plants in arid ecosystems are more, not less sensitive to drought (Knapp *et al*., 2024), there is evidence that species from warmer climates are operating closer to their upper thermal limit than cool‐climate species (Doughty *et al*., 2023) and therefore may show reduced capacity to cope with high temperatures despite traits selected for warm environments (Sage & Kubien, 2007; Clark *et al*., 2010; Aspinwall *et al*., 2019; Doughty *et al*., 2023; Choury *et al*., 2025).

**Fig. 3:**
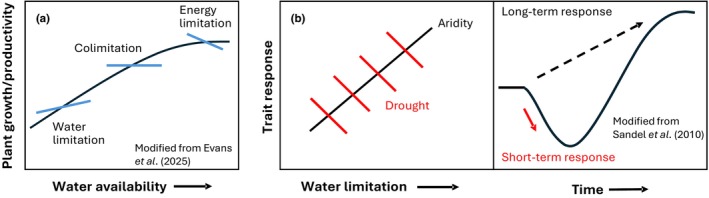
Examples of differences in spatial vs temporal responses of plants to water availability. (a) General patterns of how plant growth and ecosystem productivity may vary geographically as water availability (mean annual precipitation) increases (black line) compared with site‐based temporal relationships (blue lines, due to interannual precipitation variability) at three sites spanning arid to humid climates (after Huxman *et al*., 2004; see Evans *et al*., 2025a,b for similar relationships with temperature). Traits, if they are functionally related to growth, might be expected to respond to drought at the site level in very different ways across a spatial gradient of water availability. This is because although drought exacerbates water limitation in arid ecosystems reducing productivity from average years, its impact may be reduced in more mesic ecosystems (Knapp *et al*., 2024), and in some cases (in humid ecosystems), drought may alleviate energy (or other) limitations to growth and increase productivity (Walther *et al*., 2019; Dudney *et al*., 2023; Miller *et al*., 2023; Chen *et al*., 2025). (b) Even in more arid ecosystems, site‐level trait responses to drought (red lines) may be orthogonal to responses observed along spatial gradients of water limitation (aridity, black line). Sandel *et al*. (2010) proposed that these opposing responses are driven by different ecological mechanisms that dominate the relatively short time scales of drought (ecophysiological and initial community reordering – the red arrow in the right panel corresponds to these short‐term drought responses) vs mechanisms that require longer time scales (immigration and community turnover; Smith *et al*., 2009), and drive patterns observed along aridity gradients.

A second, related argument for the independence of aridity and drought tolerance mechanisms is that the acquisitive‐conservative framework is based on trait variation across geographic space, but drought is a temporally anomalous event (Fig. 1). Thus, an implicit space for time substitution is required to equate trait responses to aridity with drought (Fig. 3). Space for time inferences have a long history in ecology and their merits and limitations have been discussed previously (e.g. Damgaard, 2019; Lovell *et al*., 2023; Evans *et al*., 2025a,b). But differences in the causality of spatially vs temporally derived relationships and their transferability have often been noted, with a particular focus on mechanisms that differ between long vs short time scales (adaptation vs acclimation), and complicated by climate nonstationarity (e.g. Wolkovich *et al*., 2014; Liu *et al*., 2021; Felton *et al*., 2022; Griffin‐Nolan *et al*., 2023; Stemkovski *et al*., 2025; Evans *et al*., 2025a,b). For example, trait variation along geographic gradients of precipitation has been found to be opposite the short‐term trait responses to experimental manipulations of precipitation (Sandel *et al*., 2010). Transient responses of traits to drought may be driven by ecophysiological mechanisms or rapid responses in leaf traits (such as SLA) that primarily affect the extant community (Smith *et al*., 2009; W. Luo *et al*., 2023). But fundamentally different ecological mechanisms (community turnover and immigration) operate at longer time scales, potentially leading to opposing trait responses (Fig. 3; W. Luo *et al*., 2023). More broadly, while responses of ecological patterns and processes to short‐term resource/environmental manipulations can be consistent with those observed along geographic gradients or over long time scales, there is also abundant evidence that they can differ strongly in magnitude and sign relative to short‐term responses (Knapp *et al*., 2012;Elmendorf *et al*., 2015; Melillo *et al*., 2017; Yuan *et al*., 2017; Liu *et al*., 2021; Post *et al*., 2021; Ferrín *et al*., 2023; Perret *et al*., 2024). In fact, several studies suggest that traits that confer resistance to drought in the short term may be selected against in the long term (Choat *et al*., 2012; DeSoto *et al*., 2020; Tijerín‐Triviño *et al*., 2025).

Further complicating trait responses to spatial gradients in aridity and precipitation is smaller scale variability in soils. Soil texture, for example, strongly influences soil hydraulic properties, gives rise to the ‘inverse soil texture effect’ on plant water stress and ecosystem functioning across precipitation gradients, and may influence drought‐induced mortality (Noy‐Meir, 1973; Austin *et al*., 2004; Wankmüller *et al*., 2024). Drought also influences soil fungal and bacterial communities along aridity gradients in ways that differ from how plant communities are affected (e.g. Ochoa‐Hueso *et al*., 2018) potentially strengthening both positive and negative plant–soil feedback effects over time (Veresoglou *et al*., 2022). The inverse soil texture effect in particular – in which the relative degree of water limitation as determined by soil texture is the opposite in arid vs humid regions, further complicates linking spatial patterns to the temporal impacts of drought.

**Fig. 4:**
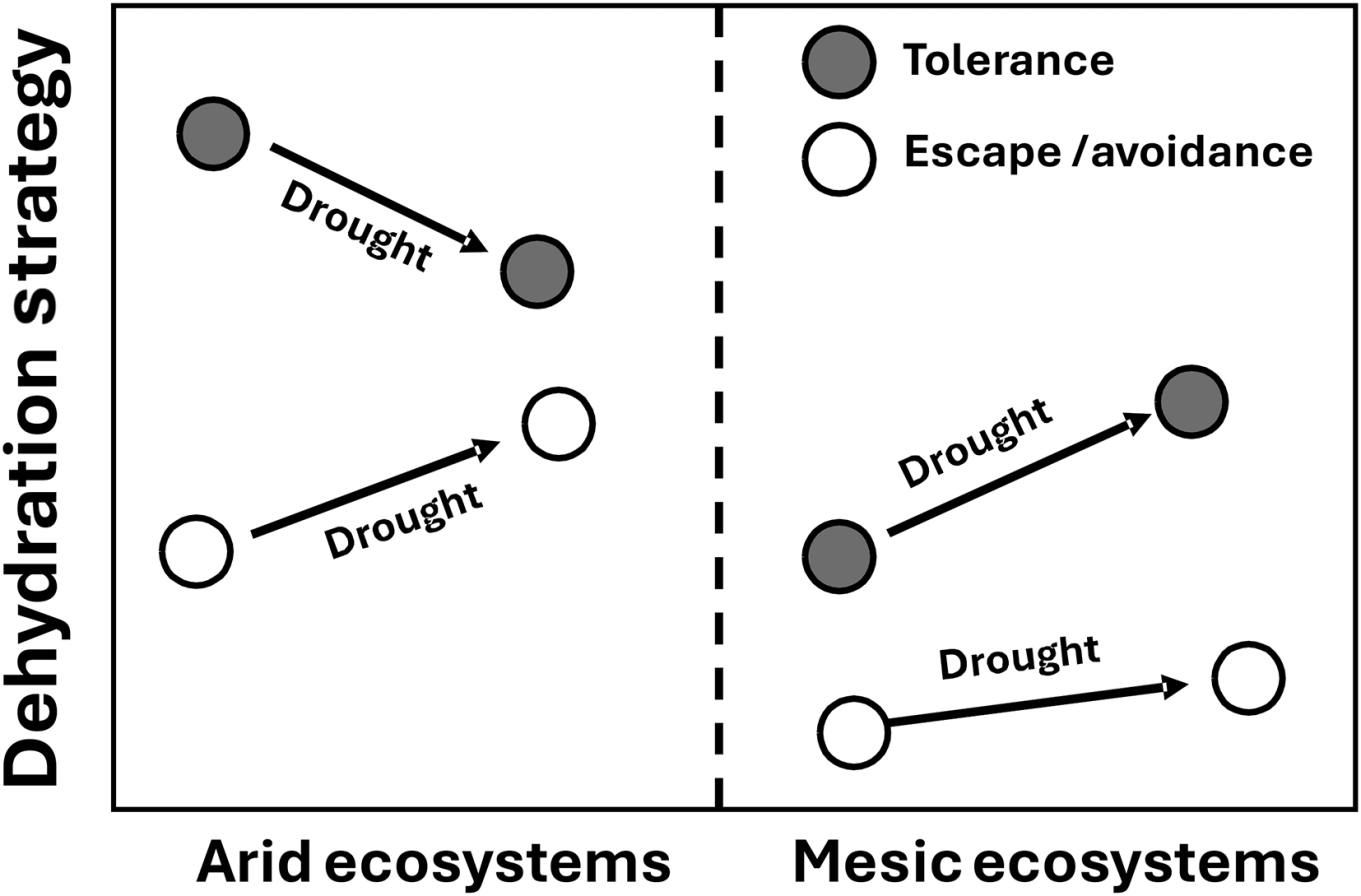
Plant traits associated with dehydration tolerance, as well as escape and avoidance strategies (Kooyers, 2015; Volaire, 2018) are generally more common in arid than mesic ecosystems. Many studies show that tolerance traits increase with drought in more mesic and humid ecosystems, consistent with trait variation observed along aridity gradients. However, in more water‐limited ecosystems, and especially in arid systems in which plants with multiple strategies co‐occur, water stress from drought may exceed the limits of dehydration tolerance, and here species with tolerance traits may decrease in response to drought, whereas plants with escape/avoidance traits increase. Thus, drought can have very different impacts on community‐weighted traits in arid vs mesic ecosystems, leading to responses that are inconsistent with how traits vary along aridity gradients.

Finally, plant strategies for coping with periods of low water availability have often been conceptualized as those that enable plants to tolerate, avoid, or escape water stress (Kooyers, 2015). While these have often been termed ‘drought strategies’, they are perhaps more correctly viewed as dehydration strategies (Volaire, 2018). Each of these strategies involve different sets of traits (Matos *et al*., 2021; Künzi *et al*., 2025), and there is growing evidence that drought interacts with these strategies differently across environmental gradients (Fig. 4). Thus, while traits conferring dehydration tolerance may generally increase from wet to dry ecosystems, escape and avoidance plant strategies are more common in dryland ecosystems. Much research has shown that when drought severity becomes extreme, the most successful plant strategies shift from tolerance to escape and avoidance in semiarid and arid ecosystems (Gross *et al*., 2013; Carvajal *et al*., 2019; Griffin‐Nolan *et al*., 2019; Knapp *et al*., 2020, 2024; Luo *et al*., 2021, W. Luo *et al*., 2023; Blanco‐Sánchez *et al*., 2022; Kramp *et al*., 2022). This can result in community‐level trait responses to drought driven by relative decreases in the abundance of species with tolerance (conservative) traits, increases in species with escape (acquisitive) strategies, and mixed responses of species with avoidance strategies depending on drought severity and timing (Forner *et al*., 2018; Carvajal *et al*., 2019; Sun *et al*., 2022; Yan *et al*., 2025). Because arid ecosystems have relatively high plant functional trait diversity and a broad range of strategies for coping with water stress (Maestre *et al*., 2021; Reich *et al*., 2026), trait responses to drought at the community‐scale may differ strongly from more mesic ecosystems that lack a similar level of functional diversity in dehydration traits.

## The way forward

While exceptions can be found to predictions arising from even the most robust ecological theories (Scheiner & Willig, 2008; Schnitzer & Carson, 2016), we posit that the inconsistent alignment of conservative‐acquisitive traits with drought responses is too widespread to be explained by the contingent nature of ecology (Lawton, 1999; Knapp & D'Avanzo, 2010). Certainly, the study of trait responses to environmental and resource gradients has been enormously successful in increasing our understanding of how plants have adapted to the array of environments present on Earth (Wright *et al*., 2004; Westoby & Wright, 2006; Díaz *et al*., 2016; Anderegg, 2023; Carmona & Beccari, 2025). But alternative frameworks (e.g. Volaire, 2018; Volaire *et al*., 2026) may be more fruitful for understanding how plants respond to drought. For example, rather than conceptually basing drought vulnerability hypotheses on how traits vary along aridity gradients, there are other frameworks that may be more mechanistically consistent based on (1) drought–trait relationships linked to hydraulic strategies (e.g. Skelton *et al*., 2015; Feng *et al*., 2019; Liang *et al*., 2026), (2) plant responses to variations in drought severity and duration (Volaire, 2018; Felton *et al*., 2021; Trugman, 2022; Knapp *et al*., 2024; Gazol *et al*., 2025), (3) differences among time scales of mechanisms that operate across the ecological hierarchy (Smith *et al*., 2009; Stemkovski *et al*., 2025), and (4) considering trait response to changes in means vs extremes independently (Sommer *et al*., 2025). For example, evaluating responses to drought and aridity within a pulse‐press framework (Bender *et al*., 1984) may be more appropriate conceptually given that different mechanisms and trait responses are expected to occur when resources are altered in the short vs long term (Smith *et al*., 2009). Indeed, viewing drought through the lens of an extreme events framework (Smith, 2011) has been argued to be essential for understanding the mechanisms driving species survival and range shifts with climate change (Soifer *et al*., 2025). Finally, recent studies suggest that drought alters biotic interactions among species (Barkaoui & Volaire, 2023) in ways that can lead to species and trait responses that are counterintuitive to expectations from aridity gradients (Osmolovsky *et al*., 2025; Ramos‐Muñoz *et al*., 2025) and that life history, phenology, and genome size may play a larger role in determining dehydration tolerance than previously suspected (Rimer & McAdam, 2025; J. Sun *et al*., 2025). These and many other drivers can modify water relations traits along the acquisitive‐conservative spectrum, as well plant responses to drought, independent of aridity.

Of the frameworks described previously, Volaire (2018) invokes both physiological and ecological mechanisms and explicitly distinguishes dehydration strategies (relevant to aridity gradients) from drought resistance and survival (Volaire *et al*., 2026). But rather than advocate for any of the frameworks mentioned previously, we argue instead that drought research underpinned by conservative vs acquisitive traits and, more broadly, by responses of plants to aridity gradients, a practice the authors of this viewpoint have themselves employed in the past (e.g. Griffin‐Nolan *et al*., 2019; W. Luo *et al*., 2023), is not likely to advance our understanding of the ecological consequences of globally increasing water limitation. This is because even when plant and trait responses to increasing aridity and drought are congruent, there is a significant probability that the mechanisms of causality differ (Stemkovski *et al*., 2025), limiting our ability to predict how these dimensions of climate change will alter ecosystem functioning in the future.

## Competing interests

None declared.

## Author contributions

AKK planned and designed the research. AC, JEG, AH, AJ, SO, JAS, JGT, EGT and RJG‐N conducted literature reviews and synthesized the literature. All authors contributed ideas and text to the manuscript and edited multiple versions. AKK and RJG‐N provided final editorial review.

## Disclaimer

The New Phytologist Foundation remains neutral with regard to jurisdictional claims in maps and in any institutional affiliations.
